# Emergence of a Clonal Lineage of Multidrug-Resistant ESBL-Producing *Salmonella* Infantis Transmitted from Broilers and Broiler Meat to Humans in Italy between 2011 and 2014

**DOI:** 10.1371/journal.pone.0144802

**Published:** 2015-12-30

**Authors:** Alessia Franco, Pimlapas Leekitcharoenphon, Fabiola Feltrin, Patricia Alba, Gessica Cordaro, Manuela Iurescia, Rita Tolli, Mario D’Incau, Monica Staffolani, Elisabetta Di Giannatale, Rene S. Hendriksen, Antonio Battisti

**Affiliations:** 1 Istituto Zooprofilattico Sperimentale del Lazio e della Toscana “M. Aleandri”, National Reference Laboratory for Antimicrobial Resistance, Via Appia Nuova 1411, 00178, Rome, Italy; 2 National Food Institute, Technical University of Denmark, WHO Collaborating Center for Antimicrobial Resistance in Food borne Pathogens, and European Union Reference Laboratory for Antimicrobial Resistance, Kgs. Lyngby, Denmark; 3 Istituto Zooprofilattico Sperimentale della Lombardia e dell’Emilia–Romagna ‘‘Bruno Ubertini”, Via Bianchi 9, 25124, Brescia, Italy; 4 Istituto Zooprofilattico Sperimentale dell’Umbria e delle Marche, Sezione di Macerata, Via dei Velini, 15, 62100, Macerata, Italy; 5 Istituto Zooprofilattico Sperimentale dell’Abruzzo e del Molise “G. Caporale”, Via Campo Boario, 64100, Teramo, Italy; Institut National de la Recherche Agronomique, FRANCE

## Abstract

We report the spread of a clone of multidrug-resistant (MDR), ESBL-producing (*bla*_CTX-M-1_) *Salmonella enterica* subsp. *enterica* serovar Infantis, in the Italian broiler chicken industry and along the food-chain. This was first detected in Italy in 2011 and led to human infection in Italy in 2013–2014.A set (n = 49) of extended-spectrum cephalosporin (ESC)-resistant (R) isolates of *S*. Infantis (2011–2014) from humans, food-producing animals and meat thereof, were studied along with a selected set of earlier and more recent ESC-susceptible (ESC-S) isolates (n = 42, 2001–2014). They were characterized by macrorestriction-PFGE analysis and genetic environment of ESC-resistance. Isolates representative of PFGE-patterns and origin were submitted to Whole Genome Sequencing. The emerging ESC-R clone, detected mainly from broiler chickens, broiler meat and humans, showed a minimum pattern of clinical resistance to cefotaxime, tetracycline, sulfonamides, and trimethoprim, beside ciprofloxacin microbiological resistance (MIC 0.25 mg/L). All isolates of this clone harbored a conjugative megaplasmid (~ 280–320 Kb), similar to that described in ESC-susceptible *S*. Infantis in Israel (pESI-like) in 2014. This megaplasmid carried the ESBL gene *bla*_CTX-M-1_, and additional genes [*tet*(A), *sul1*, *dfrA1* and *dfrA14*] mediating cefotaxime, tetracycline, sulfonamide, and trimethoprim resistance. It also contained genes conferring enhanced colonization capability, virulence (fimbriae, yersiniabactin), resistance and fitness (*qacE1*, *mer*) in the intensive-farming environment. This emerging clone of *S*. Infantis has been causing infections in humans, most likely through the broiler industry. Since *S*. Infantis is among major serovars causing human infections in Europe and is an emerging non-typhoidal *Salmonella* globally, further spread of this lineage in primary productions deserves quick and thorough risk-management strategies.

## Introduction

Infections from non-typhoidal *Salmonella* (NTS) are among the leading causes of acute enteric illness, and one of the major burdens of disease worldwide, including in the European Union [1,2]. Multidrug-resistant (MDR) *Salmonella* is associated with higher frequency and longer durations of hospitalization, prolonged illness, a higher risk of invasive infection, a two-fold increased risk in post infection morbidity, and more serious outcomes compared to infections from drug-susceptible strains [3,4,5,6].

It is estimated that invasive infections are approximately 5% of all human cases of salmonellosis [7,8]. Fluoroquinolones and third-generation cephalosporins are classified as critically important antimicrobials for human health [9], and are the drugs of choice for treating invasive infections, with the latter being the first choice for treating children, due to their pharmacodynamic properties and lack of less desirable side-effects [10]. Currently, there is an increasing global concern for the transmission of extended spectrum β-lactamase- (ESBL) and AmpC-producing *Salmonella* and *Enterobacteriaceae*, from primary production to humans [11,12]. This could potentially compromise therapeutic treatment with third-generation cephalosporins in humans, and may have serious public health implications and consequences.

*Salmonella enterica* subsp. *enterica* serovar Infantis is emerging worldwide [13]. It is the most frequently reported serovar in broilers (26%), the second most prominent one in broiler meat (37.4%) and the fourth most prevalent one in NTS human infections in Europe [14]. Over the last decade, MDR *S*. Infantis has increasingly been reported in Italy from food-animals and humans [15], and is also highly prevalent in the broiler meat industry, in several European countries [16].

In addition, human cases of extended-spectrum cephalosporin (ESC)-resistant *S*. Infantis have been detected in Belgium and France, which directly disseminated from poultry via the IncI1 plasmid carrying the ESBL gene *bla*_TEM-52_ [17].

Recently, there has been an increase in MDR and ESC-resistant (ESC-R) *Salmonella* isolates detected in samples from broiler chicken flocks in Italy [11,16]. According to the Italian antimicrobial resistance (AMR) monitoring data on isolates from the National Control Program (NCP) in broiler chicken flocks in 2012 and 2013, ESC-resistance rates in *Salmonella* spp. reached 15.6% (12/77, 95% CI 8.3–25.6%) and 20.3% (13/64, 95% CI 11.3–32.2%), respectively, of which most were *S*. Infantis. Over the same period, MDR ESC-R *S*. Infantis was often detected in humans, in Italy.

The aim of the study was to elucidate the linkage and transmission of ESC-R *S*. Infantis in Italy along the food chain; from poultry, meat, and other primary production systems to human clinical cases. We investigated these aspects using a variety of molecular methods, including, end-point PCRs, Pulsed Field Gel Electrophoresis (PFGE), plasmid characterization, microarrays, and whole genome sequencing, to assess clonal relatedness as a tool for source attribution and prevention purposes.

## Materials and Methods

### Isolates

*Salmonella* Infantis isolates (n = 91) from national AMR monitoring activities conducted from 2001 to 2014 by the National Reference Laboratory for Antimicrobial Resistance, the Istituto Zooprofilattico Sperimentale del Lazio e della Toscana (IZSLT), Rome, Italy, were included in this study. From a total of n = 49 ESC-R (2011–2014), *S*. Infantis isolates, 23 were from different broiler chicken flocks (18 from national control programs, 3 from laboratory-based surveillance, 2 from national monitoring), 17 from unrelated human clinical cases, six from broiler meat, two from pork, and one from a pig holding. In addition, an outgroup of n = 42 ESC-susceptible (ESC-S) *S*. Infantis isolates from 2001 to 2014 was randomly selected from *Salmonella* isolates from national AMR monitoring activities: 6 from different broiler chicken flocks (4 from national control programs, 2 from laboratory-based surveillance), 22 from unrelated human clinical cases, six from broiler meat, five from pork, 2 from “unspecified” meat, and one from a guinea fowl holding. The details on isolates included in this study are available in Table A in S1 File.

### Antimicrobial susceptibility testing

Antimicrobial susceptibility testing was performed as minimum inhibitory concentrations (MIC) using micro-broth dilutions in 96-well microtitre plates (Trek Diagnostic Systems, Westlake, OH, USA). The following antimicrobials were tested: ampicillin (AMP), cefotaxime (CTX), ceftazidime (CAZ) ciprofloxacin (CIP), chloramphenicol (CHL), gentamicin (GEN), nalidixic acid (NAL), sulfamethoxazole (SMX), tetracycline (TET), and trimethoprim (TMP). The results were interpreted according to the European Committee on Antibiotic Susceptibility Testing (EUCAST) epidemiological cut-offs (www.eucast.org). EUCAST clinical breakpoints were used for those drugs where epidemiological cut-offs were unavailable: kanamycin, chloramphenicol, sulfamethoxazole, trimethoprim. For streptomycin, a cut-off of 16 mg/L was used, according to EUCAST MIC distributions [18]. *Escherichia coli* ATCC 25922 was used was used as a Quality Control strain.

Phenotypic confirmatory tests for the detection of ESBLs were performed on 49 isolates resistant to cefotaxime or ceftazidime according to Clinical Laboratory Standard Institute (CLSI) recommendations [19]. *Klebsiella pneumoniae* ATCC 700603 was used as a Quality Control strain.

### Detection of genes encoding beta-lactamases, carbapenemases and plasmid-mediated quinolone resistance

PCR was used to test the 49 confirmed ESBL-producing isolates for genes encoding beta-lactamases (*bla*_CTX-M_, *bla*_SHV_, *bla*_TEM,_ *bla*_OXA_, *bla*_CMY-1_, *bla*_CMY-2_), plasmid-mediated quinolone resistance (PMQR: *qnrA*, *qnrS*, *qnr*B, *qnr*D, *qep-*A, *aac(6')-Ib-cr*), and carbapenemases (*bla*_(IMP)_, *bla*_(VIM)_, *bla*_(NDM)_, *bla*_(KPC)_ and *bla*_(OXA-48)_), as previously described [20]. Amplicons were Sanger sequenced by BigDye Terminator chemistry (Applied Biosystems, Foster City, CA, USA) using an automated sequencer; ABI Prism 310; Applied Biosystems. Sequence data analysis was performed using CLC DNA workbench software version 5.7.1 (CLC Bio, Aarhus, Denmark) and evaluated against the GenBank nucleotide database (http://www.ncbi.nlm.nih.gov/nucleotide/).

### Plasmids and genetic environment of ESC-resistance

#### Identification and characterization of plasmids

The detection and identification of plasmid replicons in all isolates under study was performed by PCR-based replicon typing using the PBRT kit (Diatheva, Fano, Italy), as previously described [21,22].

For plasmid size estimation, S1 nuclease PFGE was applied [23]. PFGE plugs of 69 isolates, either ESC-R or ESC-S, containing the intact genome were incubated with S1 nuclease for 2h at 37°C. PFGE was performed, using a CHEF-DRII (Bio-Rad Laboratories GmbH, Munich, Germany) with the following run conditions: 22h, 6V/cm, with an initial pulse of 2.2s and a final pulse of 63.8s. Plasmid sizes were estimated using ImageLab software (BioRad, Hercule CA, USA) with the *Salmonella enterica* serotype Braenderup H9812, restriction digested with XbaI enzyme, as a size marker.

The presence of selected pESI-like plasmid sequences/fragments in 60 isolates (encoding for plasmid backbone; *mer* operon; yersiniabactin siderophore system; two novel chaperon-usher fimbriae *ipf* and *K88*-like) were investigated using a set of end-point PCRs with primers and conditions (Table B in S1 File), as previously described. This approach also allowed screening for the presence of the pESI-like plasmid in the isolates [24].

To demonstrate the presence and location of the ESBL gene, *bla*_CTX-M-1_ harbored by the pESI-like plasmid, the DNA fragment based on the S1 nuclease PFGE plugs (bands of ~ 280–320 kb) was extracted and purified with the QIAquick Gel Extraction Kit (Qiagen, Hilden, Germany). End-point PCR for the *bla*_CTX-M_ gene group and Sanger sequencing of the amplicon were performed as described above.

#### Conjugation experiment

To demonstrate plasmid transferability by conjugation, cultures of the donor *S*. Infantis 12034722/1, 12037823/11, 1302124/34, 14957027/15, and recipient *Escherichia coli* (*E*. *coli*) K12 were mated by mixing overnight cultures in Luria-Bertani broth in 100 and 300 μL volumes, respectively. The cultures were incubated overnight at 22°C, 30°C and 37°C. Subsequently, the overnight cultures were plated on MacConkey agar containing rifampicin (100 μg/mL) and cefotaxime (1μg/mL) to select transconjugants.

Transconjugants were screened for plasmid size, presence of IncP replicon, *bla*_CTX-M-1_ gene, and marker genes of the pESI-like plasmid found in the donor, as described above. Resistance genes in the plasmid of transconjugants were screened by microarray using the AMR-ve Genotyping Kit (Alere Technologies, GmbH, Germany).

### Macrorestriction PFGE

Macrorestriction analysis was conducted by digesting the PFGE plugs with the *Xba*I enzyme, according to a slightly modified PulseNet protocol (http://www.cdc.gov/pulsenet), using the same instrument and run conditions as those for the S1 nuclease PFGE [23]. Cluster analysis was performed using the BioNumerics version 7 software (Applied Maths, Sint-Martens-Latem, Belgium), and a dendrogram was created applying the Dice Similarity coefficient with an optimization and tolerance of 1.5% each, and clustering with the UPGMA method. *Salmonella enterica* serotype Braenderup H9812 strain was used as the molecular size marker [25].

### Whole Genome Sequencing and bioinformatics tools

A representative subset of 12 isolates was whole genome sequenced on the basis of PFGE clustering, source, combination of pESI-like and *bla*_CTX-M_ genes, and the presence of other plasmid replicons. Genomic DNA was extracted from the isolates using an Invitrogen Easy-DNA^TM^ Kit (Invitrogen, Carlsbad, CA, USA) and DNA concentrations were determined using the Qubit dsDNA BR assay kit (Invitrogen). The genomic DNA was prepared for Illumina pair-end sequencing using the Illumina (Illumina, Inc., San Diego, CA) NexteraXT® Guide 150319425031942 following the protocol revision C (http://support.illumina.com/downloads/nextera_xt_sample_preparation_guide_15031942.html). A sample of the pooled NexteraXT Libraries was loaded onto an Illumina MiSeq reagent cartridge using MiSeq Reagent Kit v2 and 500 cycles with a Standard Flow Cell. The libraries were sequenced using an Illumina platform and MiSeq Control Software 2.3.0.3. The 12 isolates were pair-end sequenced.

Raw sequence data have been submitted to the European Nucleotide Archive (http://www.ebi.ac.uk/ena) under study accession no.: PRJEB10819 (http://www.ebi.ac.uk/ena/data/view/PRJEB10819). The raw reads were assembled using the Assembler pipeline (version 1.0) available from the Center for Genomic Epidemiology (CGE) http://cge.cbs.dtu.dk/services/all.php, which is based on the Velvet algorithms for *de novo* short reads assembly. The complete list of genomic sequence data is available in Table C in S1 File. The assembled sequences were analyzed to confirm the species and *Salmonella* serotype using the CGE pipelines: K-merFinder (version 2) and SeqSero (version 1.1). Following confirmation, the MLST sequence type (ST) for *Salmonella enterica*, plasmid replicons, and acquired AMR genes were identified using the pipelines: MLST (version 1.7), PlasmidFinder (version 1.2), and ResFinder (version 2.1) with 98% identity and 60% minimum alignment length as thresholds, also available from the CGE.

Whole genome shotgun sequences of *S*. Infantis str. 119944 plasmid pESI were retrieved from GenBank with the following accession numbers: ASRF01000099—ASRF01000108. Sequence of *E*.*coli* plasmid beta-lactamase *bla*_CTX-M-1_ gene, with accession number DQ915955, was downloaded from GenBank. In order to examine the presence of pESI-like plasmid containing the *bla*_CTX-M-1_ gene, the assembled genomes of *S*. Infantis were aligned against the *bla*_CTX-M-1_ gene using BLASTN. The contig of *S*. Infantis containing the *bla*_CTX-M-1_ gene was aligned against the pESI-like plasmid using BLASTN to check its presence in the contig.

SNPs were determined using the CSI Phylogeny 1.0a [26] pipeline available from the CGE website. Basically, each of the raw reads were aligned against the reference *S*. Infantis SINFA (accession number; LN649235) genome using Burrows-Wheeler Aligner (BWA) version 0.7.2 [27]. SNPs were called using ‘mpileup’ module in SAMTools version 0.1.18 [28]. Subsequently, SNPs were selected if they met the following criteria: (i) a minimum distance of 15 base pairs (bp) between each SNP, (ii) a minimum of 10% of the average depth, (iii) the mapping quality was greater than 30, (iv) the SNP quality was greater than 20 and (v) all indels were excluded. The qualified SNPs from each genome were concatenated to a single alignment corresponding to a position of the reference genome, using an in-house Perl script. In cases where SNPs were absent in the reference genome, they were interpreted as not being a variation, and the base from the reference genome was included [29]. The concatenated sequences were subjected to multiple alignments using MUSCLE from MEGA5 [30]. The final phylogenetic SNP tree was computed by MEGA5 using the maximum-likelihood method of 1,000 bootstrap replicates.

## Results

### Antimicrobial susceptibility testing

**Fig 1:**
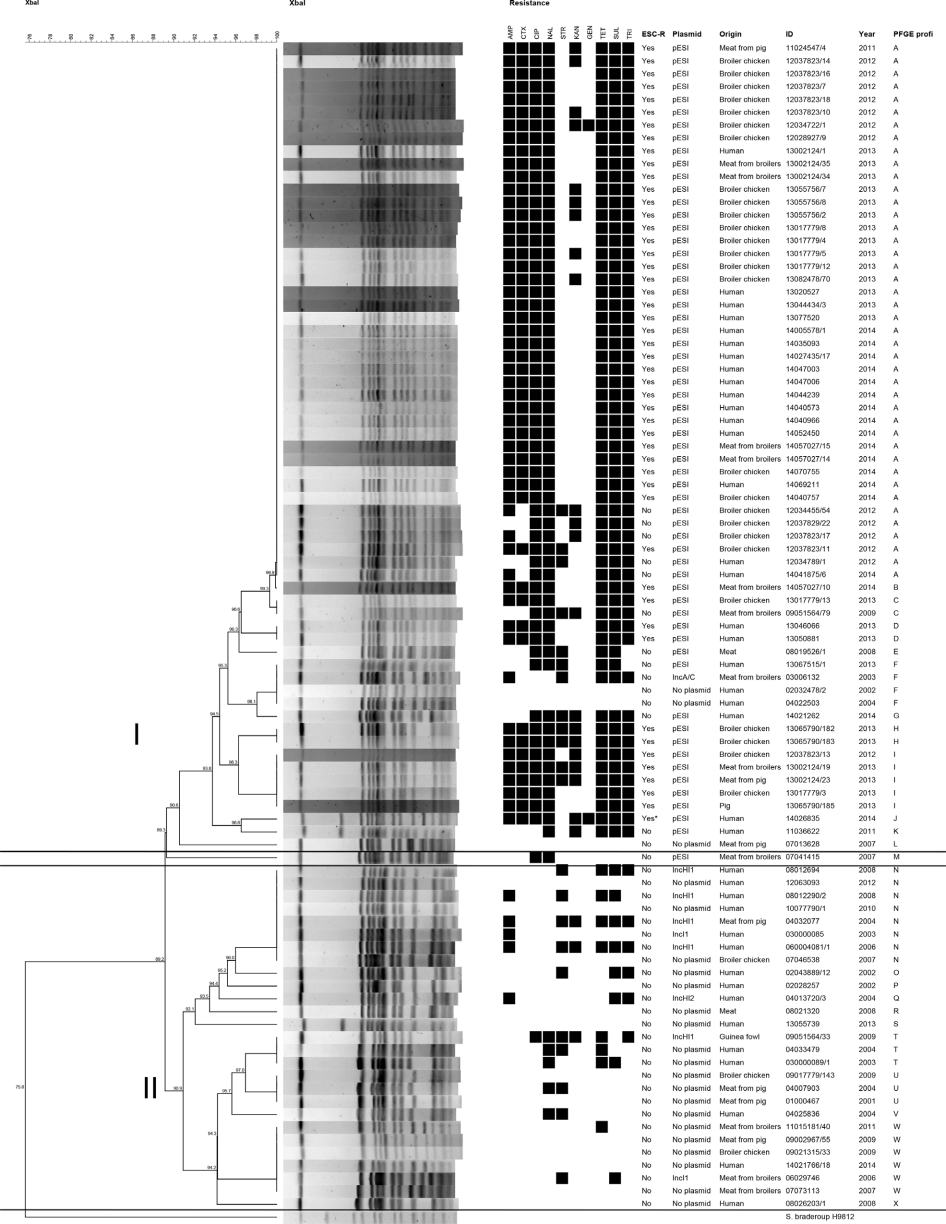
XbaI PFGE macrorestriction cluster analysis and antimicrobial resistance patterns of 91 ESC-susceptible and ESC-resistant Salmonella Infantis (ST32) from humans, animals and meats thereof, 2001–2014. *Abbreviations*: ESC-R: Extended-spectrum cephalosporin resistance; AMP: ampicillin; CTX: cefotaxime; CHL: chloramphenicol; CIP: ciprofloxacin; NAL: nalidixic acid; STR: streptomycin; KAN: kanamycin; GEN: gentamicin; TET: tetracycline; SMX: sulfamethoxazole; TMP: trimethoprim

MDR was a constant feature of the ESC-R isolates, with a minimum common resistance pattern of CTX-[AMP]-TET-SMX-TMP-NAL-CIP. MICs to CTX (8 mg/L), AMP (64–128 mg/L), TET (128 mg/L), SMX (2048 mg/L), TMP (64 mg/L) were all also in the range of clinical resistance according to the EUCAST Standard, along with NAL (128 mg/L), and CIP (0.25 mg/L) microbiological resistance. None showed a concurrent phenotype of resistance to carbapenems. The complete results of resistance patterns in all isolates under study are shown in Fig 1 and in Table A in S1 File.

### Genes encoding ESC-resistance and PMQR

All ESC-resistant *S*. Infantis investigated showed the presence of only the *bla*_CTX-M-1_ gene, except for the human isolate 14026835 which, in contrast, carried the *bla*_CTX-M-65_ gene. All isolates tested negative for carbapenemase genes and PMQR determinants.

### Genetic environment of ESC-resistance

#### PCR-based Replicon Typing (PBRT) and WGS results

The sixty isolates, including the 49 ESC-R isolates, tested positive for the IncP incompatibility group using PBRT, and were confirmed to solely harbor a pESI-like megaplasmid of around 280–320 Kb. In addition, the WGS analysis of the 12 isolates confirmed that the pESI-like plasmid contained the bla_CTX-M-1_ or the bla_CTX-M-65_ genes in 8 isolates; 14026835, 13065790/185, 13002124/1, 14057027/15, 13002124/34, 13017779/5, 14035093, 12037823/11, because the bla_CTX-M_ gene and the pESI-like plasmid were present in the same contig, and the alignment positions of the bla_CTX-M_ gene were in-between the alignment positions of the plasmid (Table D in S1 File).

#### pESI-like plasmid characterization

In all pESI-like positive isolates, the IncI1 pMLST replicase gene *repl1* was always absent, in contrast to the *ardA* gene, which was always present (Table A in S1 File).

In the subset of the 12 isolates investigated by WGS, IncI1 pMLST in the pESI-like plasmid was positive for three or four genes only (*ardA*, *pilL*, *sogS*, *trbA*), (Table E in S1 File), and was always negative for the replicase gene *repl1*. The IncI1 alleles in the ESC-S isolates and the CTX-M-1-pESI-like plasmids were *ardA*_2, *pilL*_3, *sogS*_9, *trbA*_21, except one, which had a truncated a*rdA* allele (22/343 HSP, 14057027–15, broiler meat), and the earliest (2007) documented ESC-S-pESI-like, which was negative for the a*rdA* and the *piL* alleles (19/254 HSP, 07041415, broiler meat). In contrast, the *bla*_CTX-M-65_-pESI-like plasmid carried *ardA*_11, *pilL*_3, *sogS*_14, *trbA*_8 (Table E in S1 File).

All the *bla*_CTX-M_-pESI-like plasmids, carried the AMR resistance genes, *tet*(A), *sul1*, and *dfrA1* (mediating resistance to TET, SMX, and TMP, respectively), while 3/8 also carried the *aadA1* gene (streptomycin/spectinomycin resistance). All, except one (14057027/15, broiler meat), also carried the *dfrA14* gene. The *bla*_CTX-M-65_-pESI-like plasmid carried the *dfrA1*, and was negative for the *dfrA14* (Table A in S1 File).

**Fig 2:**
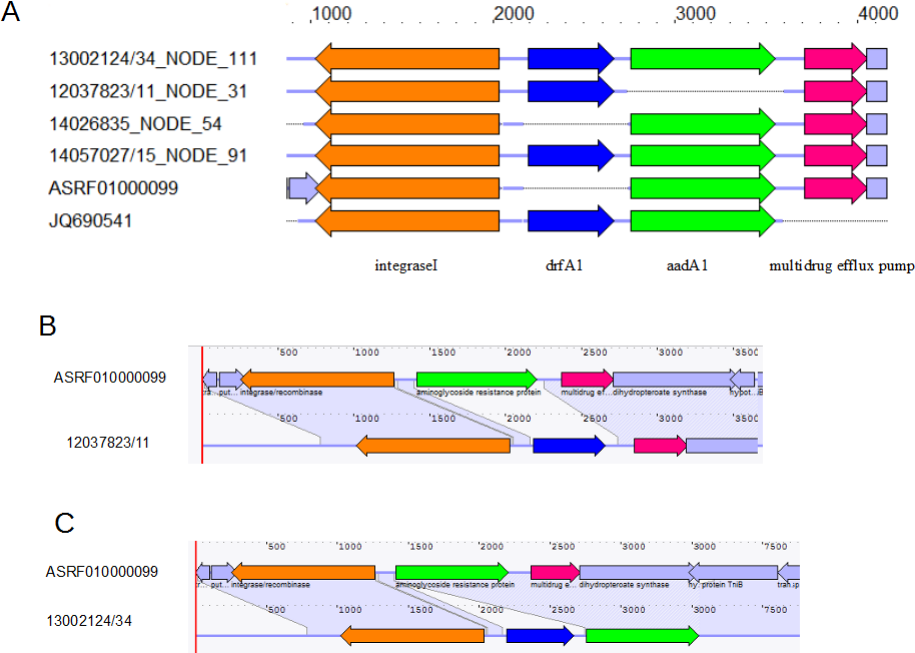
Different combinations of antimicrobial resistance genes in the same region of pESI-like plasmids harbored by *Salmonella* Infantis. A) Multiplex alignment of the region where *drfA1* and *aadA1* genes are located in the pESI-like plasmid, in different combinations, in the isolates of this study; in the pESI plasmid (ASRF01000099); and in the reference sequence for the *drfA1* gene (JQ690541). The arrows represent the genes: integrase I (orange), *drfA1* (blue), *aadA1* (green), multidrug efflux pump (pink). B) Pairwise comparison of the pESI-like megaplasmid in the 12037823/11 isolate, and the pESI plasmid (ASRF01000099) where the “substitution” of *aadA1* by *drfA1* in the isolate of this study is represented. C) Pairwise comparison of the pESI-like megaplasmid in the 13002124/34 isolate, and the pESI megaplasmid (ASRF01000099) where the “insertion” of *drfA1* in the isolate of this study is represented.

In the Italian megaplasmids, *dfrA1* either replaced the *aadA1* gene, or flanked it. Fig 2 shows examples of differences in the integration of AMR resistance genes found in some pESI-like Italian megaplasmids, compared to the Israeli pESI.

All the pESI-like plasmids were positive for genes or fragments investigated by PCR (backbone, *mer* operon, yersiniabactin, *K88-like* fimbria *fim*). In the 12 selected isolates, WGS further confirmed the presence of genes associated with virulence or enhanced fitness, in the pESI-like plasmid (genes confirmed included the yersiniabactin biosynthetic protein gene *irp*, the Infantis plasmid-encoded fimbria gene *ipf*, the *K88*-like usher *feaD*, and *qacE1*). These results are reported in Table F in S1 File.

Finally, among ESC-S isolates under study, three isolates carried the plasmid replicon IncI1, four isolates carried the IncHI1, one isolate carried the IncA/C, and one isolate carried the IncHI2. The two incI1 plasmids (from broiler meat, 2006, and guinea fowl, 2009) submitted to MLST, were both ST26 (Table E in S1 File).

The complete set of plasmids detected and their sizes in all isolates studied are reported in Table A in S1 File.

### Conjugation experiment

The pESI-like plasmids in all donor strains tested were successfully transferred to the recipient E. coli K12 strain, with a frequency of 8.3*10^-10 (estimated for the isolate 12037823/11).

The pESI-like plasmids detected in the *bla*_CTX-M-1_-positive transconjugants were also positive for *tet*(A), *sul1*, *dfrA1* and *dfrA14*, and *aadA1* mediating resistance to TET, SMX, and TMP, and STR, respectively, in the same combination as present in the donor strains (Table G in S1 File)

### Macrorestriction PFGE cluster analysis, MLST and Phylogenetic SNP analysis

#### Macrorestriction PFGE cluster analysis

The PFGE analysis using XbaI endonuclease revealed 24 unique PFGE patterns, which were separated by > 90% similarity into two defined PFGE groups I and II, as well as one more distantly located isolate. Group I included 63 out of 91 (69.2%) isolates, of which 42 (46% of the total) had indistinguishable profiles (pulsotype A). Pulsotype A was mainly composed (37/42, 95.2%) of pESI-like-positive, ESC-R, ESBL-producing organisms, isolated from broiler chickens, broiler meat and humans, except one isolate which was from pork, and were all detected from 2011 to 2014. All but one pESI-like-positive isolates, were included in group I. Indeed, pESI-like-positive, ESC-R isolates represented more than three-fourths of this cluster (48/63, 77.4%).

The isolates of group II were all ESC-S, harbored different plasmids or were plasmid-free and were more heterogeneous, even though some profiles were repeated: eight isolates shared pulsotype N, six shared pulsotype W, three pulsotype T and three pulsotype U. These PFGE-indistinguishable isolates were obtained from humans, broilers, pigs, or meats thereof, and in one case (pulsotype T) from guinea fowl and humans. Most strains in group II were isolated earlier (2002–2010) than those in group I, with 25/29 (86%) detected before 2011.

The distant isolate ESC-S, pESI-like-positive 07041415 shared an 89.3% similarity with isolates from groups I and II.

#### MLST and Phylogenetic SNP analysis

All isolates submitted to WGS belonged to the Sequence Type (ST) ST32, except one human isolate (13001224–34), which was a single-locus variant (SLV, *hisd*) of ST32.

**Fig 3:**
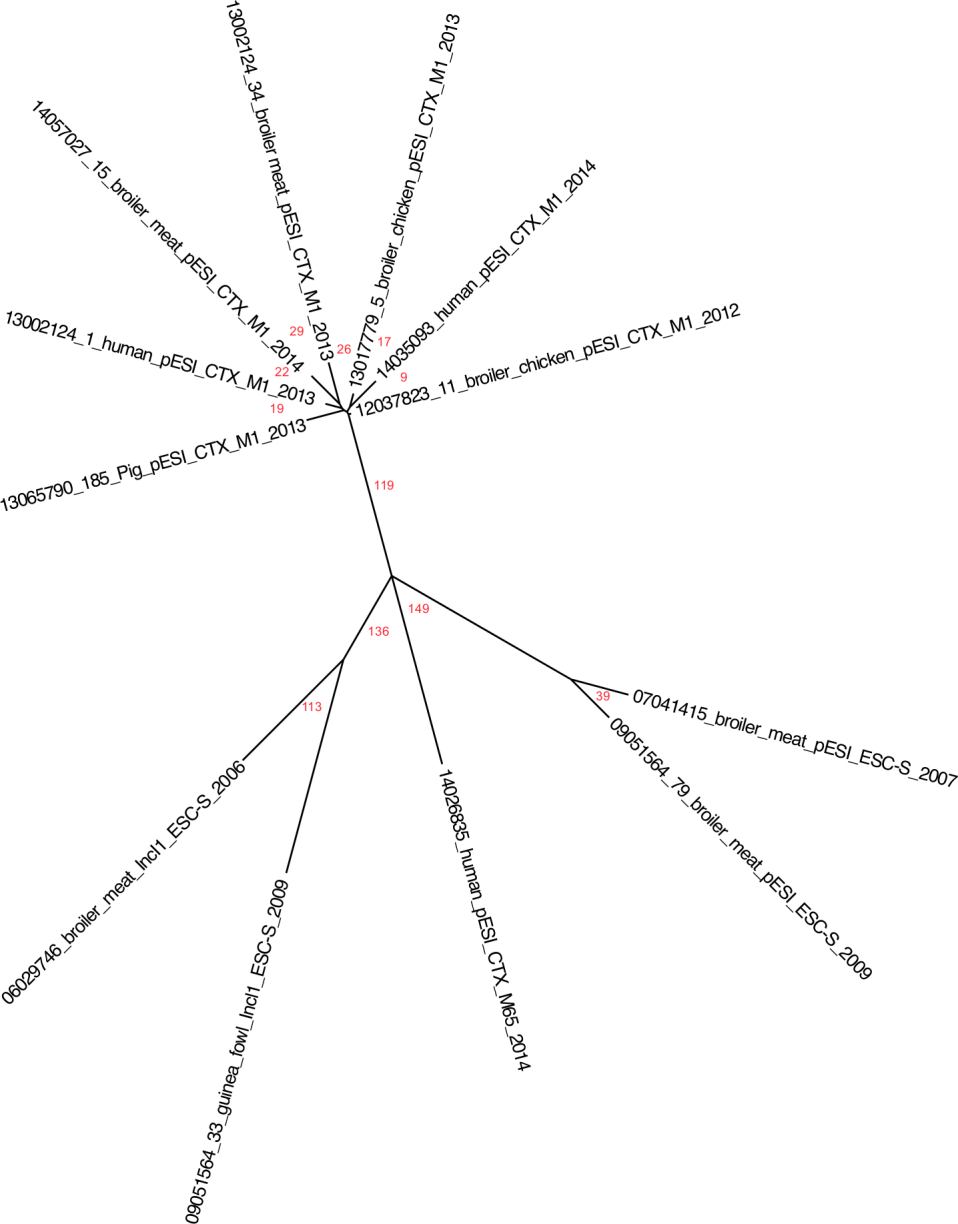
Single-nucleotide polymorphism (SNP)-based phylogeny of 12 selected ESC-resistant and ESC-susceptible *Salmonella* Infantis from poultry, meats, and humans, in Italy (2006–2014).

Genomic relatedness was analyzed by mapping the 12 genomes to the reference genome, *S*. Infantis SINFA. An unrooted phylogenetic SNP tree was generated based on a total of 547 informative SNPs, and it is shown in Fig 3.

Seven of the 12 genomes were grouped in one distinct phylogenetic cluster, and included the almost contemporary pESI-like-positive, *bla*_CTX-M-1_-positive human, poultry and pig isolates only (n = 7), separated by 119 SNPs from the remaining five isolates. These latter five isolates consisted of: two previously isolated ones (2007 and 2009), ESC-S broiler isolates containing the pESI-like megaplasmid, the human isolate (14026835), which carried the *bla*_CTX-M-65_ gene, and two other previously detected (2006 and 2009) ESC-S isolates containing the IncI1 plasmid replicon. All genomes in the cluster described above were relative clonal, linked by only nine to 29 SNPs. This supports the PFGE analysis where all strains of this cluster were also clustered by PFGE in group I and were designated pulsotype A except for the pig isolate 13065790/185, which belonged to pulsotype I within the same group I and had a similarity of 94.5% to pulsotype A.

As for the remaining five isolates, the two ESC-S broiler isolates containing the pESI-like megaplasmid, were more distantly related than the cluster isolates, but were pairwise separated by only 39 SNPs, in contrast to the PFGE analysis. Indeed, strain 07041415 in the PFGE dendrogram was displaced between PFGE group I and II, and designated pulsotype M, whereas 09051564/79 belonged to PFGE group I with pulsotype C (Figs 1 and 3). The two previously detected ESC-S isolates, containing the IncI1 plasmid replicon, seemed more closely related compared to the other isolates, being separated by 113 SNPs. Both of these isolates belonged to PFGE group II, but were categorized into different pulsotypes (94.2% similarity). The human isolate (14026835), which carried the *bla*_CTX-M-65_ gene, radiated on the phylogenetic tree by itself and differed from the two ESC-S isolates containing the pESI-like megaplasmid by 149 SNPs, and from the other two ESC-S isolates containing the IncI1 plasmid replicon, by 136 SNPs (Fig 3).

## Discussion

This study provides the first evidence for the emergence of an MDR, ESBL-producing *S*. Infantis clone harboring a conjugative pESI-like megaplasmid containing the ESBL gene *bla*_CTX-M-1_. The *S*. Infantis clone was mostly detected in the Italian broiler chicken industry, from where it disseminated through the food chain to humans.

A mosaic-megaplasmid designated pESI (approx. 280 Kb), which was recently reported in Israel [24], conferred resistance to an ESC-S *S*. Infantis, boosted its pathogenicity and led to increased intestinal inflammation in experimental mouse infections, in comparison to the plasmid-free ancestral strain. The Israeli pESI also harbored genes conferring resistance to TET, SMX, and TMP, and increased bacterial tolerance to environmental mercury (*mer* operon) and oxidative stress. The authors concluded that pESI is a chimeric plasmid that evolved by recombination between, at least, IncI1 and IncP ancestral plasmid groups [24].

In this study, the vast majority of isolates segregated into PFGE group I, and all isolates in pulsotype A, most of which proved to be ESC-R, and harbored a similar megaplasmid, designated pESI-like, of approximately 280–320 kb. An exception was a single distant ESC-S isolate, 07041415 from broiler meat, which did not belong to the two PFGE groups. This strain was also distantly displaced in the SNP analysis, together with another ESC-S broiler meat isolate, 09051564/79. The vast majority of pESI-like-positive, ESC-R isolates in this PFGE group I were detected in broiler chicken holdings, broiler meat or humans from 2011 to 2014. This group is composed of a main cluster of isolates that appear indistinguishable by PFGE cluster analysis (PFGE profile A), and represent a clonal lineage in the SNP analysis where isolates were separated by nine to 29 SNPs, thus indicating clonal relation [31].

Interestingly, five isolates (3 from broilers and 2 from humans) within this cluster harbor the pESI-like plasmid, but were ESC-S. The single distant ESC-S isolate from broiler meat, sharing 89% similarity with this main PFGE group I, provides evidence for the presence of this megaplasmid in *Salmonella enterica* serovars in Italy as early as 2007.

Interestingly, all the alleles of the IncI1 group identified in this early pESI-like chimeric plasmid were the same as those found in broilers, broiler meat and humans in the main pulsotype A, and in general, in the PFGE group I. Additionally, the specific combination of these four (or three) alleles was also never described in any IncI1 previously. Conversely, the “true” IncI1 plasmids of this study differed in the content of all alleles (Table E in S1 File), and belonged to ST26 (CC26).

Substantial agreement with the PFGE cluster analysis was obtained in the SNP phylogenetic tree constructed on selected isolates that were whole genome sequenced. Here, the human isolate carrying the *bla*_CTX-M-9_-group gene *bla*_CTX-M-65_ on a pESI-like plasmid was the only ESBL-positive isolate out-grouped with ESC-S isolates, detected previously (2006–2009). All IncI1 alleles of pMLST, except for *piL*_3, were also different in this isolate compared to other pESI-like plasmids of PFGE group I (Table E in S1 File). This feature may be a consequence of the independent acquisition of a different pESI-like plasmid by the “earlier clone” of *S*. Infantis, also harboring a different ESBL gene.

Indeed, this *bla*_CTX-M-65_-positive isolate also carried the *fosA* gene, but lacked the 16S rRNA methylase genes (e. g. *rmtB* and *armA*), often found in plasmids of the IncFII incompatibility groups in the dominant *E*. *coli* of animal origin from China [32,33].

Taken together, the results of our study are in agreement with the mosaic nature previously proposed for the Israeli pESI megaplasmid, since we also found a pMLST profile in common (except for the replicase gene *repl1*) with the incompatibility plasmid groups IncI1 and the concurrent presence of conserved IncP replicon sequences. It is likely that the similarities between the two plasmids may be due to the existence of a common ancestor, but could potentially also be explained by common selection pressure factors in similar intensive poultry farm settings, leading to similar outcomes in the evolution of these plasmids. However, further studies are required to clarify these aspects.

Interestingly, both IncP and IncI1 were already detected in Italy in earlier ESC-S *S*. Infantis isolates (until the year 2008), although at that time, IncH1 was the dominant one [15]. A gradual acquisition of resistance genes by the pESI-like may have occurred over the years, possibly through insertion and deletion by transposition mechanisms (Fig 2) [34]; for instance, none of these genes were detected in the 2007 isolate (07041415) and only two out of four genes (*tet*(A) and *sul1*) were found in the 2009 isolate, both from broiler meat. Conversely, more recently, *bla*_CTX-M-1_-positive pESI-like plasmid in the SNP tree cluster carried all the four resistance genes, with all also carrying *dfrA1*, in addition to the *drfA14* found in the Israeli isolate. The AMR gene content in the Italian pESI-like differs from the Israeli pESI, in which only the *dfrA14* was present (str. 119944, GenBank accession n. ASRF01000000), and which also carried the *aadA1* gene. Moreover, other differences were found between the two plasmids, in the region where the *aadA1* gene was located in the Israeli pESI. Specifically, the presence of *aadA1* is variable in the Italian megaplasmids as it is sometimes substituted by the *drfA1* gene (Fig 2).

The plasmid is likely to have acquired the *bla*_CTX-M-1_ gene from commensal *E*. *coli* populations of the intestinal microbiota of Italian broiler chickens, in which it represents the most common ESBL(data not shown).

Similar to the pESI-positive *S*. Infantis from Israel, this Italian emerging clone had additional genes encoding for factors associated with increased ability for colonization and virulence (e. g. fimbriae or the yersiniabactin siderophore system), along with those associated with resistance and enhanced fitness (e. g. *qacEΔ1*, *mer* operon), which may be advantageous in the intensive-farming environment. Some of these genes can also be considered targets for applied research supporting vaccine-based control strategies.

Most importantly, our study provides information on the capability of this emerging clone to also acquire plasmid-borne ESBL genes, such as the *bla*_CTX-M-1_ gene, which not only confer traits mediating increased pathogenicity, but also a resistance trait of major concern. Another trait of concern is reduced susceptibility (MIC 0.25 mg/L) to fluoroquinolones, a major class of critically important antimicrobials (CIAs) for human therapy, since elevated ciprofloxacin MICs (> 0.125 mg/L) in typhoidal and non-typhoidal *Salmonella* are associated with poor response and increased probability of treatment failure [35,36].

On average, the isolates of this emerging *S*. Infantis clone were resistant to a higher number of antimicrobials. Besides resistances mediated by genes on the pESI-like plasmid, a significantly higher proportion of CIP (and NAL) microbiologically resistant isolates is evident in the PFGE pulsotype A, in the SNP cluster, and in general in PFGE group I, as compared to group II.

Once this clone acquired *bla*_CTX-M-1_ and became ESC-resistant, it most likely disseminated among broiler flocks by further clonal spread, which was probably also favored by the pyramidal structure of this food-producing animal industry. However, the conjugative nature of the megaplasmid may favor its horizontal spread in other Enterobacteriaceae and *Salmonella*, and its further success in intensive farming environments. A crucial role for its emergence and possible future spread is likely to be played by the selection pressure with aminopenicillin beta-lactams (e. g. ampicillin, amoxicillin), possible off-label use of third- or fourth-generation cephalosporins (e. g. ceftiofur), and the co-selection exerted by the use of other drug classes, mainly tetracyclines, sulfonamides, trimethoprim (fluoro)quinolones, and aminoglycosides, considering the multidrug-resistant nature of this clone (Fig 1). Noteworthy, in Italy, third- and fourth- generation cephalosporins have never been licensed for use in poultry, and off-label use is currently prohibited, according to EU legislation.

Since, in recent years, the reported percentage of ESC-resistant *Salmonella* spp. from the Italian national control program in broiler chicken flocks exceeded 15% (most of which are *S*. Infantis), while ESC-R prevalence in pig holdings and pork meat is very low, it is reasonable to assume that most exposure for humans comes from poultry meat. Although the earliest ESBL (CTX-M1-pESI-like-positive) *S*. Infantis in our study is from pork meat and dates back to 2011, all other isolates are from broiler chickens (or meat thereof) and from humans, and have been increasingly detected from 2012 onwards. The only exception is a pig isolate detected in 2013.

Interestingly, the pESI-like-positive, ESC-S isolates, sharing high similarity with the ESBL-producing clone, were still found in broiler chickens and humans in these last two years (four isolates of broiler chicken origin, and four from humans, respectively in 2012–2014), although they are less represented in the collection used for this study.

In conclusion, an emerging ESBL-producing, MDR *S*. Infantis clone has been recently spreading in Italy, mostly in the broiler chicken industry, and is causing infections in humans along the food chain, most likely through broiler meat, as demonstrated by PFGE cluster analysis and WGS SNP phylogeny. Resistance to extended-spectrum cephalosporins, in a multidrug-resistant clone also showing reduced susceptibility to fluoroquinolones, is *per se* a matter of great concern. Indeed, although this emerging clone with enhanced virulence traits still remains susceptible to last resort drugs like carbapenems, its genetic background drastically reduces the spectrum of therapeutic options available to humans, especially in the case of invasive infections.

However, since *S*. Infantis is one of the major serovars causing human infections in Europe and an emerging non-typhoidal *Salmonella* worldwide, the spread of this clone in the food-producing animal system, and especially in the broiler chicken industry, deserves quick and thorough risk-management strategies, for actions to be taken with input and collaboration from all stakeholders.

## Supporting Information

S1 File Table A: Metadata, plasmid molecular weights and selected associated genes, antimicrobial susceptibility, and antimicrobial resistance genes in 91 ESC-resistant and ESC-susceptible *Salmonella* Infantis, Italy, 2001–2014. Table B: Primers and conditions for selected PCRs targeting pESI-like plasmid genes/fragments and IncI1 pMLST genes. Table C: List of genomic sequence data of the 12 *Salmonella* Infantis isolates submitted for Whole Genome Sequencing. Table D: Alignment positions of *bla*_CTX-M_ genes within the pESI plasmids of the *Salmonella* Infantis isolates submitted for Whole Genome Sequencing. Table E: IncI1 alleles in the pESI-like megaplasmid and in the two IncI1 plasmids harbored in the 12 *Salmonella Infantis* isolates submitted for Whole Genome Sequencing. Table F: Details of other selected colonization, virulence, and resistance genes within the pESI-like plasmids of the *Salmonella Infantis* isolates submitted for Whole Genome Sequencing. Table G: Antimicrobial resistance genes on the pESI-like plasmid transferred by conjugation from *Salmonella* Infantis to the recipient *Escherichia coli* K12 strain. (XLS)
